# Targeting neuroplasticity in patients with neurodegenerative diseases using brain stimulation techniques

**DOI:** 10.1186/s40035-020-00224-z

**Published:** 2020-12-07

**Authors:** Ti-Fei Yuan, Wei-Guang Li, Chencheng Zhang, Hongjiang Wei, Suya Sun, Nan-Jie Xu, Jun Liu, Tian-Le Xu

**Affiliations:** 1 Shanghai Key Laboratory of Psychotic Disorders, Shanghai Mental Health Center, Shanghai Jiao Tong University School of Medicine, Shanghai, 200030 China; 2 Co-Innovation Center of Neuroregeneration, Nantong University, Nantong, Jiangsu 226001 China; 3 Center for Brain Science, Shanghai Children’s Medical Center, and Department of Anatomy and Physiology, Shanghai Jiao Tong University School of Medicine, Shanghai, 200025 China; 4 Department of Functional Neurosurgery, Ruijin Hospital, Shanghai Jiao Tong University School of Medicine, Shanghai, 200025 China; 5 Institute for Medical Imaging Technology, School of Biomedical Engineering, Shanghai Jiao Tong University, Shanghai, 200030 China; 6 Department of Neurology and Institute of Neurology, Ruijin Hospital, Shanghai Jiao Tong University School of Medicine, Shanghai, 200025 China

**Keywords:** Alzheimer’s disease, Parkinson’s disease, Synapse, Neurotransmitter, Synaptic plasticity, Brain stimulation, Deep brain stimulation, Transcranial magnetic stimulation

## Abstract

Deficits in synaptic transmission and plasticity are thought to contribute to the pathophysiology of Alzheimer’s disease (AD) and Parkinson’s disease (PD). Several brain stimulation techniques are currently available to assess or modulate human neuroplasticity, which could offer clinically useful interventions as well as quantitative diagnostic and prognostic biomarkers. In this review, we discuss several brain stimulation techniques, with a special emphasis on transcranial magnetic stimulation and deep brain stimulation (DBS), and review the results of clinical studies that applied these techniques to examine or modulate impaired neuroplasticity at the local and network levels in patients with AD or PD. The impaired neuroplasticity can be detected in patients at the earlier and later stages of both neurodegenerative diseases. However, current brain stimulation techniques, with a notable exception of DBS for PD treatment, cannot serve as adequate clinical tools to assist in the diagnosis, treatment, or prognosis of individual patients with AD or PD. Targeting the impaired neuroplasticity with improved brain stimulation techniques could offer a powerful novel approach for the treatment of AD and PD.

## Background

Alzheimer’s disease (AD) and Parkinson’s disease (PD) are common neurodegenerative disorders characterized by a progressive decline in cognitive and motor functions, respectively. Both disorders are associated with neuronal loss in various brain regions, particularly the hippocampus associated with memory impairment in AD [1] and the substantia nigra pars compacta associated with motor dysfunction in PD [2]. Impaired synaptic plasticity in affected brain structures and networks is thought to represent a critical pathological mechanism underlying the progressive cognitive and motor deficits seen in these neurodegenerative disorders [3, 4].

Synaptic plasticity involves a complex series of presynaptic and postsynaptic biochemical events that are triggered by external or internal stimuli and may induce short- or long-standing changes in the strength of synaptic transmission, thereby modifying brain structure and function, and subsequently, behavior [5]. Persistent and activity-dependent strengthening (termed long-term potentiation; LTP) and weakening (long-term depression; LTD) of excitatory synapses in the hippocampus are widely thought to underlie the learning and memory processes in the mammalian brain. Although the precise electrical and chemical events responsible for the modification of synaptic strength remain poorly understood, it seems that both the presynaptic release of glutamate and the activation of *N*-methyl-*D*-aspartate receptors are required for the initiation of subsequent biochemical processes that give rise to LTP or LTD in the hippocampal memory-related circuits [5]. Persistent forms of synaptic plasticity like those found in the hippocampus have been identified in other brain areas and networks, including the dopaminergic nigrostriatal pathway, which has been implicated in the pathogenesis of PD and the progressive decline of motor functions in PD patients, including the impaired motor skill learning [2, 3].

The impaired synaptic plasticity thus may be a basic cellular mechanism mediating the progressive cognitive and motor deficits observed in AD and PD patients. If this hypothesis were valid, measures of human brain synaptic plasticity and its impairment could offer vital quantitative biomarkers that could aid in the diagnosis and prognosis of patients with AD or PD [6, 7]. Moreover, therapeutic modulation of the impaired synaptic plasticity in affected patients, e.g., using brain stimulation or neuropharmacological interventions, would be expected to alleviate, delay, or halt the progressive clinical deterioration seen in these disorders [4, 6].

To date, most evidence supporting the hypothesis that the impaired synaptic plasticity contributes to the progressive cognitive and motor deficits in AD and PD has come from cellular and animal models, as well as from post-mortem neuropathological studies in brain tissues of patients. For example, in the context of the amyloid hypothesis of AD, amyloid precursor protein transgenic mice have been found to display impaired in vitro and in vivo LTP in the hippocampus, which correlates with the spatial memory deficits [8]. Similarly, in the 6-hydroxydopamine rat model of PD, striatal LTP and LTD were found to be aberrant, whereas chronic treatment with the dopamine precursor levodopa (*L*-dopa) restored the deficits in striatal synaptic plasticity [3].

However, the findings from animal research and human postmortem neuropathological studies cannot be readily generalized to the brain and cognitive functions and dysfunctions in living persons. Fortunately, the past two decades have witnessed the development of various noninvasive and invasive brain stimulation techniques that permit the measurement or modulation of synaptic plasticity in the living human brain. These novel brain stimulation techniques, ranging from transcranial magnetic stimulation (TMS) [9] to deep brain stimulation (DBS) [10], allow for the implementation of neural stimulation systems with unprecedented spatial and temporal precision. Here, we first discuss the different brain stimulation techniques currently available and then evaluate the results of clinical studies that applied these techniques to assess or modulate the impaired neuroplasticity at the local and network levels in AD and PD patients.

## Main text

In the past decade, various noninvasive and invasive brain stimulation techniques have been utilized to measure and/or modulate impaired neurotransmission and plasticity in patients with AD or PD. The noninvasive brain stimulation techniques used include TMS, transcranial direct current stimulation (tDCS), transcranial alternating current stimulation (tACS), and transcranial ultrasound stimulation. In several studies, these noninvasive brain stimulation techniques have been found to improve the cognitive deficits in AD [11] and the motor symptoms of PD [12, 13]. The invasive brain stimulation techniques employed include intracranial recordings of local field potentials (LFPs) and associated neuronal oscillations in different frequency bands, DBS of the subthalamic nucleus (STN) or globus pallidus internus (GPi) in patients with PD [10, 14] and the recent DBS of the fornix white matter bundle in patients with AD [15]. Among the brain stimulation techniques, DBS is a well-established effective tool in the clinical management of patients with movement disorders, including PD [10, 12, 13, 16]. Here, we mainly focus on TMS and DBS, which are used in many clinical studies published so far, as well as tDCS, which has often been used in studies of patients with PD.

### TMS

**Fig. 1:**
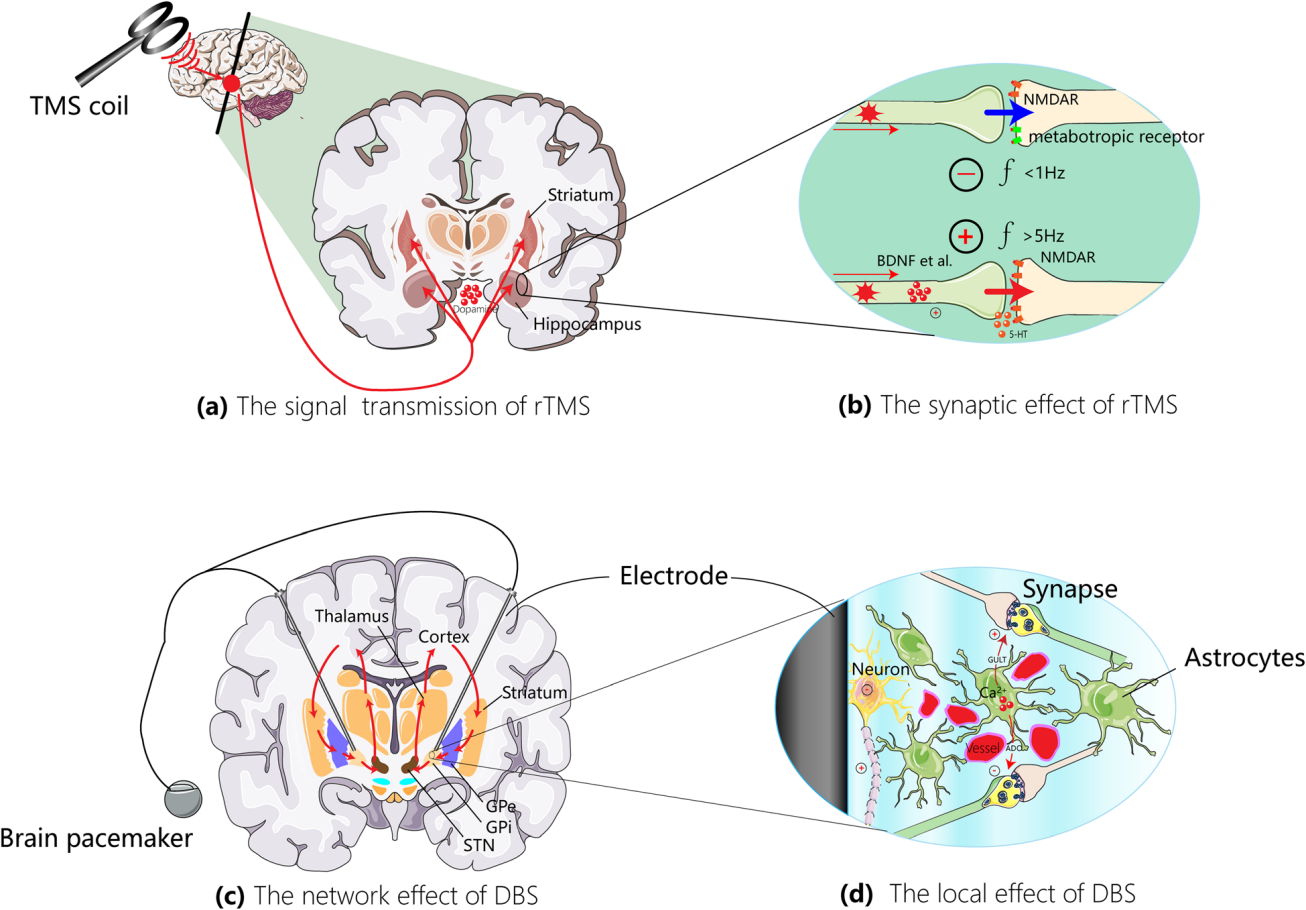
The effects of DBS and rTMS in the brain. **a** Basic principles of rTMS and its network effects. The TMS involves the delivery of a transient magnetic field through a coil placed on the surface of the skull, thereby producing a brief electrical current that activates a small area of brain beneath the coil. While the delivery of a single TMS pulse can transiently activate or inhibit the underlying cortical region, that of rTMS pulses can induce longer-lasting, plasticity-like changes in brain functions. It is commonly assumed that the rTMS-induced cortical plasticity and network activation are responsible for its actions on motor and cognitive function and dysfunction. Typically, cortical rTMS can evoke striatal dopamine release (see red arrows), which in turn results in changes of cortical plasticity. Please see the text for more details. **b** Synaptic modulation effects of rTMS. The rTMS can modulate NMDAR and/or metabotropic glutamate receptor (mGluR)-dependent synaptic plasticity probably by enhancing the release of different neurotransmitters (i.e. glutamate, GABA), modulating glial activity, promoting neurotrophic signaling (i.e., BDNF), and promoting calcium-mediated signaling, thereby influencing synaptic transmission even in distal brain regions. **c** Basic principles of DBS. The DBS involves the delivery of electric current to an electrode implanted in a brain structure or nucleus of interest. The effects of DBS can be influenced by the brain tissue surrounding the DBS electrode and the spatial configuration of activated or inhibited neuronal populations in the target brain structure. The physiological effects of DBS are complex and can occur at the molecular, cellular, local, and network levels. Of note, the inherent complexity and wide range of effects of DBS can extend beyond the target network and function of interest. Moreover, DBS has lasting effects on neurotransmitter concentration, function, dynamics, and glial activity, thereby altering the microenvironment of the brain and influencing neural plasticity. Red arrows denote presumable signal flows under STN DBS in PD patients. Please see the text for more details. **d** The local cellular effects of DBS include the inhibition of neuronal-cell bodies and the activation of neighboring axons as well as astrocytes. Abbreviations: DA, dopamine; f, frequency; NMDAR, *N*-methyl-D-aspartic acid receptor; mGluR, metabotropic receptor; BDNF, brain-derived neurotrophic factor; 5-HT, serotonin; GPe, globus pallidus externus; GPi, globus pallidus internus; STN, subthalamic nucleus; GLU, glutamate; ADE, adenosine

TMS involves the delivery of a transient magnetic field through a coil placed on the surface of the skull, thereby producing a brief electrical current that activates a small area of brain beneath the coil [9] (Fig. 1a). TMS can easily be combined with structural brain MRI for TMS targeting, with simultaneous scalp EEG or EMG recordings, and with associated motor-evoked potentials (MEPs), which are focal surface muscle twitches following a brief TMS pulse above the motor cortex. TMS-evoked potentials, which are time- and phase-locked to the onset of the TMS pulse itself, can also be extracted from the scalp EEG. The delivery of a single TMS pulse can transiently activate or inhibit the underlying cortical region, while the delivery of repetitive TMS (rTMS) pulses can induce longer-lasting, plasticity-like changes in brain functions [9]. In past decade, researchers have found that the delivery of 3-pulse 50-Hz bursts at a frequency of 5 Hz, referred to as the theta burst stimulation (TBS), induces levels of cortical plasticity similar to those produced using conventional rTMS protocols [17].

The paired-pulse TMS could be used as a measurement tool for cortical functioning. Short intracortical inhibition (SICI) and short intracortical facilitation (SICF) are common measures used in the paired-pulse TMS studies, which are based on the MEP amplitude evoked by a test stimulus presented at a short latency after the delivery of an initial conditioning stimulus. SICI typically occurs at latency intervals less than 5 ms after the onset of the test stimulus, whereas SICF emerges at intervals between 8 and 30 ms. It is thought that SICI reflects GABAergic, especially the GABA-A-mediated interneuron inhibition in the cortex [9]. Another commonly used measure involves the threshold for producing an MEP response, which appears to be affected by drugs targeting voltage-dependent sodium or calcium channels [9]. In paired-associative stimulation (PAS) studies, the TMS measures of interest are usually short-afferent inhibition (SAI) and long-afferent inhibition (LAI), which are elicited at latencies of about 20 ms and 200 ms, respectively, after somatosensory stimulation of the hand or peripheral nerve electric stimulation. SAI is believed to reflect the sensory-motor plasticity in the motor cortex and seems to be mediated mainly by muscarinic acetylcholine receptors [18].

The neurobiological mechanisms through which rTMS impacts brain function in health and disease are not yet fully understood. It is commonly assumed that the rTMS-induced cortical plasticity and network activation are responsible for its action on motor and cognitive function and dysfunction [19] (Fig. 1a). It has been demonstrated that rTMS influences remote brain regions, enhances the release of different neurotransmitters, modulates glial activity, and promotes neurotrophic signaling [20–22] (Fig. 1b). Also, rTMS stimulation seems to evoke glutamate/GABA release [23, 24] and to facilitate calcium-mediated signaling, thereby modulating synaptic plasticity [25] (Fig. 1b). In addition, cortical rTMS can evoke striatal dopamine release and is able to induce changes in cortical plasticity (Fig. 1a).

In general, the TMS approach has been found useful in assessing the excitability in specific cortical regions and in mapping different sensory, cognitive, and motor functions [9]. rTMS is also effective to briefly facilitate or inhibit brain and cognitive functions in patients with neurodegenerative diseases, but whether it could facilitate cognitive functions in healthy persons remains controversial [26].

### TMS in AD

#### Measurement studies

To assess the functional integrity of the primary motor cortex in AD, an early study evaluated the MEP-based SAI in 15 patients with AD and 12 age-matched healthy controls [27]. The results showed that the SAI size was significantly reduced compared with that of the healthy controls. Furthermore, administration of a single dose of the cholinesterase inhibitor rivastigmine increased the SAI in a subgroup of 6 patients. The authors suggested that SAI could serve as a noninvasive test to assess cholinergic transmission and sensory-motor plasticity in the motor cortex of patients with AD. Subsequent rTMS studies have confirmed and extended these results [18, 27–32]. For example, one study demonstrated the early occurrences of impaired SAI and MEP amplitudes in AD [18]. Another study reported that patients with AD displayed reduced motor thresholds and MEP onset latencies, which correlated with the AD symptom severity [29]. Furthermore, the reduced motor thresholds in AD patients do not seem to correlate with the impaired inhibitory effects on cortical neurons, as measured by SAI and SICI [30]. Another study using intermittent TBS has demonstrated that the dopaminergic pathways are also involved in the cortical plasticity in AD by showing that the impaired LTP-like cortical plasticity in affected patients could be restored by administration of the dopamine agonist rotigotine [33]. In addition, the TMS-based measures of LTP-like cortical plasticity seem to have predictive value for cognitive decline, even for the rate of decline, in patients with AD [34]. Although the LTD types of cortical plasticity are typically not impaired in patients with AD [35], the dopaminergic modulation of LTD-like plasticity induced by low-frequency (1 Hz) rTMS stimulation has been reported to be impaired in patients with AD, which could be restored by means of levodopa treatment [36]. Taken together, these findings indicate that the impaired sensory-motor plasticity and hyperexcitability of the motor cortex are independent contributors to, or are the consequences of, the primary pathophysiological processes that give rise to AD.

More recently, the TMS-based measurements have also been found useful in differentiating patients with AD from patients with frontotemporal dementia or dementia with Lewy bodies [37–39]. If these findings are confirmed, TMS parameters may be developed into clinically useful biomarkers that can help improve the diagnostic accuracy and differential diagnosis of AD [34, 40].

#### Treatment studies

Most studies on rTMS treatment in AD patients have focused on the dorsolateral prefrontal cortex (DLPFC) due to its involvement in cognitive functions, particularly working memory and executive behavioral control [41]. To assess the DLPFC plasticity in AD patients, one study used a PAS procedure involving trains of low-frequency (0.1 Hz) TMS pulses applied to the DLPFC combined with scalp EEG recordings and median nerve electric stimulation at the wrist [42]. After the PAS procedure, the participants also completed a cognitive task assessing the working memory. The results showed that the PAS-induced potentiation of cortical, TMS-evoked potential recorded over the DLPFC was significantly smaller in patients with AD than in age-matched healthy controls. The patients also performed more poorly in the working memory task than healthy controls. Moreover, the extent of PAS-induced long-term type of potentiation in the DLPFC was associated with the performance in the working memory task. These results have been substantiated and generalized to the population of patients with mild cognitive impairment [11, 43–46]. These findings suggest that the dysfunction of DLPFC and working memory impairment are an early pathophysiological and cognitive feature of AD.

A randomized, sham-controlled rTMS study reported that five daily sessions of high-frequency (20 Hz) rTMS over the DLPFC improved cognitive functioning, daily living activities, and mood/depressive symptoms in patients with mild to moderate AD, which were maintained at 1- and 3-month follow-up [47]. By contrast, the low-frequency (1 Hz) rTMS did not yield significant clinical benefits to patients in this study. Furthermore, a sham-controlled tDCS study found that the daily at-home tDCS over the DLPFC for 6 months improved or stabilized cognitive function and the rate of regional cerebral glucose metabolism in 11 patients with AD [48]. These results indicate that the rTMS- or tDCS-based interventions could play an important role in AD treatment, but the findings were preliminary and tentative due to the small sample size and limited experimental control.

In addition, it has been reported that the cognitive dysfunction in patients with AD could be predicted from the measures of long-distance functional connectivity (derived from the TMS-EEG-evoked component P30 generated in the parietal cortex) between the DLPFC and the superior parietal cortex [49]. Similarly, several other studies [50–53] have found that rTMS applied to the frontal, temporal, or parietal cortical regions can improve the memory, attention, and language abilities in patients with mild to moderate degrees of AD, but again it remains to be established whether these improvements are robust and can be sustained over the long-term course of AD [54].

Cognitive training interventions have been developed that can improve the cognitive function in mild to moderate stages of AD [55–57], and the combination of these interventions with rTMS may yield larger and synergistic effects on clinical symptoms of patients. To test this, a small study interlaced rTMS with daily cognitive training sessions for 6 weeks, followed by maintenance sessions for an additional 3 months, in patients with probable AD, treated for more than 2 months with cholinesterase inhibitors [58]. The results showed that the combination of rTMS with cognitive training yielded significant improvements in the cognitive functioning and daily living activities of patients at 6-week and 4.5-month follow-ups. A multicenter randomized, double-blind, sham-controlled study (*n* = 131 at study entry, *n* = 129 at follow-up) substantiated that the combination of cognitive training with rTMS yielded improvements in cognitive function in 60- to 90-year-old, unmedicated patients with mild AD [59]. These findings suggest that the combination of rTMS with cognitive training could be a valuable approach to AD treatment. As discussed later, the combination of rTMS with physical therapy may be similarly beneficial for patients with PD.

### TMS in PD

The administration of rTMS over the primary motor cortex or DLPFC has been found to improve the motor symptoms and non-motor symptoms (e.g., cognitive deficits, and affective symptoms) in patients with PD [12, 13, 60]. Several rTMS studies have assessed the excitability and plasticity of the motor cortex in patients with PD. A paired-pulse study examined SICI and SICF in 12 PD patients at both ON and OFF medication states and in 12 age-matched healthy controls [61]. The results revealed that SICF was increased in the PD patients in the OFF-medication state and was reduced by the administration of dopaminergic medications. Furthermore, the reduction in SICF from the OFF- to ON-medication state correlated with the improvement in PD motor signs. By contrast, SICI was found to be reduced in the PD OFF-state and could only be partially normalized by dopaminergic medications. The authors suggest that PD patients may be characterized by abnormally increased facilitation of certain cortical motor circuits, as well as by abnormally decreased inhibition of motor cortex activity. In addition, a recent study found reduced thresholds for producing MEPs in patients with PD dementia, which were also detected in AD and vascular dementia [62], indicating that the hyperexcitability of the motor cortex, as indexed by MEP-based motor thresholds, may not be specific to PD or AD.

Another study used a PAS protocol to examine the MEP-based cortical plasticity in 16 patients with moderate PD and 9 healthy controls [63]. The results showed that the PAS increased the MEP size in healthy controls but not in patients who were off medication. Moreover, *L*-dopa restored the deficit in the PAS-induced MEP potentiation in one subgroup of 7 patients defined by the presence of dyskinesias, while it failed to restore the MEP-potentiation deficit in the other subgroup of 9 patients with dyskinesias [63]. Similar supporting evidence for the aberrant motor cortex plasticity in PD has been reported by another study using PAS [64] and a study using intermittent TBS [65]. Interestingly, in PD patients treated with DBS of the STN, the PAS-induced cortical plasticity was only evident when both DBS and medication were ON [66], indicating that DBS combined with medication can reverse the impairment of PAS-induced motor cortex plasticity in PD patients.

Several studies have used tDCS to assess cortical plasticity in PD. When tDCS is used, the person under study is required to wear a headgear containing electrodes through which current can be delivered. Like rTMS, prolonged (e.g., several minutes) tDCS administration results in changes in cortical excitability that outlast the period of stimulation [67]. The administration of so-called anodal tDCS makes the brain more active and responsive, whereas cathodal tDCS decreases the activity and has inhibitory effects. It is assumed that the cortical plasticity induced by tDCS is mediated by changes in neurotransmitter function, neurotrophic signaling, and glial activity [68–70]. Clinical studies have reported that anodal tDCS over the DLPFC improves cognitive function in PD patients [48, 71, 72], as well as improving their motor functions when applied to the cortical motor areas [72, 73]. Notably, anodal tDCS combined with rTMS has been found to exert interactive, synergistic facilitating effects on gait function of PD patients [74]. Similarly, anodal tDCS combined with physical therapy seems to produce larger improvements of gait and balance in PD patients than using either tDCS or physical therapy alone [75].

### DBS

DBS involves the delivery of electric current to an electrode implanted in a brain structure or nucleus of interest, such as the STN in PD (Fig. 1c). The physiological effects of DBS vary by stimulation parameters (e.g., frequency, amplitude, pulse width and duration), DBS target of interest, and the preexisting brain state. In addition, the DBS effects can be affected by the brain tissue surrounding the DBS electrode, as well as by the spatial configuration of neuronal populations activated or inhibited in the targeted brain structure [76]. The physiological effects of DBS are complex and can occur at the molecular, cellular, local, and network levels (Fig. 1c) [10, 77]. Furthermore, it is important to know the inherent complexity and widespread effects of DBS, which can extend beyond the targeted networks and functions of interest (Fig. 1c) [76]. DBS has persisting effects on neurotransmitter concentration, function, and dynamics, as well as on glial activity, thereby changing the microenvironment of brain and affecting the neuroplasticity (Fig. 1d) [78, 79].

It should be added that the neurosurgical implantation of DBS electrodes provides unique opportunities to record LFPs near the contact point. Time-frequency analysis of the LFP data makes it possible to assess the integrity of neuronal oscillations in different frequency bands. The neuronal oscillations observed at the LFP level are not necessarily locally generated but may reflect the temporal summation and ‘integration’ of activity from spatially distinct populations of neurons. This allows investigation of neural synchrony by applying short trains of high-frequency DBS to induce or modulate neuronal oscillations. For example, high-frequency DBS of the STN produces an enduring LFP-based potentiation in the substantia nigra pars reticulata of patients who have received an oral administration of *L*-dopa, whereas the patients who have not received *L*-dopa administration do not show an enduring potentiation [80]. These results demonstrate that DBS can be a valuable tool to examine and modulate neuronal oscillations, which are considered to be the basis for higher brain and motor functions.

### DBS in AD

DBS has revolutionized the treatment and care of PD patients over the past three decades [10, 81], but the application of DBS for the management of cognitive impairment in AD has only been in the beginning. Studies of DBS treatment have mainly focused on the functional integrity of the fornix in AD patients. The fornix is the major white matter fiber bundle in the limbic system and forms important input and output pathways of the hippocampus, a brain region known to mediate learning and memory processes. Accordingly, fornix DBS is hypothesized to improve memory function in AD by modulating dysfunctional hippocampal memory circuits and networks. A randomized, sham-controlled, double-blind clinical trial, however, found no significant changes in cognitive function at 1-year follow-up in patients with mild AD who had received fornix DBS [82]. In another randomized clinical trial, fornix DBS did not affect the cognitive outcomes of AD patients (*n* = 42), although the stimulation occasionally triggered spontaneous memory flashbacks in 48% of the patients during the initial programming of the stimulator [83]. The recollection of these vivid memories of past events reflects the declarative long-term memory, or episodic memory, which is known to be mediated by hippocampal networks and disrupted in AD. It remains to be determined why the fornix DBS treatment failed to affect the memory function in the AD patients in these two studies.

A possible explanation is that these studies used open-loop DBS, rather than the closed-loop DBS that can provide timely stimulation in response to the pathological brain activity [15]. Compared to the open-loop DBS, the closed-loop DBS is more sensitive and more powerful, because the programming of DBS parameters is conducted automatically based on the measured biomarker. Indeed, it has been proposed that the disruption of intracranial LFPs or associated fast neuronal oscillations may be a rapid and effective feedback signal in the closed-loop DBS treatment for AD [15].

### DBS in PD

As mentioned above, DBS of the STN or GPi is a safe and effective treatment for motor symptoms of PD, but the therapeutic mechanisms remain elusive. It is commonly assumed that DBS improves PD symptoms and signs by restoring abnormal dopaminergic neurotransmission and synaptic plasticity in motor structures and networks in affected patients [10, 66, 76]. Yet, the modulation of dysfunctional glutamatergic and GABAergic pathways within the thalamocortical and corticostriatal networks may also contribute to the clinically significant improvements in motor and non-motor symptoms of severely affected, medication-refractory patients receiving DBS of the STN or GPi [10, 76, 84].

Additional clinical evidence for the involvement of neuroplasticity facilitation in the therapeutic effects of DBS in PD has come from the observation that the symptoms of PD respond to DBS treatment on dramatically varied timescales (Table 1). Most commonly, tremor and rigidity are alleviated rapidly (within seconds or minutes) after DBS, possibly through its immediate action on aberrant neurotransmission and network motor function. It takes more time (e.g., hours) for the improvement of bradykinesia by DBS, which may stem from the short-term changes in synaptic transmission and plasticity. Finally, it takes even more time (days or weeks) for axial signs of PD to respond to DBS, indicating the involvement of more enduring changes in the brain, especially the long-term plasticity and ultimately functional reorganization (Table 1).

**Table 1:** ^a^Time course of clinical effects and hypothesized therapeutic mechanisms of DBS in PD [77, 85]

Mechanism	Time after turning DBS on	PD symptom
Immediate modulation of synaptic function	Seconds	Tremor
		Rigidity
	Minutes	Tremor
		Rigidity
		Bradykinesia
Short-term synaptic plasticity	Hours	Bradykinesia
		Axial symptoms
Long-term synaptic plasticity (functional reorganization)	Days	Axial symptoms
	Weeks	Axial symptoms

^a^Based on refs [77, 85]

### Future directions

Various noninvasive and invasive brain stimulation techniques have emerged as valuable tools for the assessment of brain plasticity and functional modulation of cognitive and motor networks in health and disease. However, apart from DBS that has proven effective for PD, extensive research efforts are still required before these brain stimulation tools can be applied to the clinical management of neurodegenerative diseases such as AD and PD. As indicated above, a promising area of further research is the combination of different brain stimulation tools, or the combination of a single brain stimulation tool with cognitive training in AD or with physical therapy in PD. Further development of closed-loop DBS is expected to offer a powerful clinical tool that is faster and more effective in restoring ongoing pathological brain activities, especially in AD.

In addition, the use of PAS typically involves the pairing of motor cortex TMS pulses with peripheral sensory nerve stimulation. A recent study employed a new technical protocol and reported that the pairing of DBS pulses at the STN and TMS pulses at the primary motor cortex at specific time intervals can induce cortical plasticity in PD patients [86]. This combination of rTMS and DBS offers a new tool to assess and modulate cortical plasticity in patients with neurodegenerative diseases. Similarly, further development of ultrasound stimulation [87] may become another brain stimulation tool to examine and modulate the impaired synaptic transmission and plasticity in neurodegenerative diseases.

In a similar vein, a recent animal study on addiction used low-frequency DBS of the nucleus accumbens paired with a dopamine receptor D1 antagonist to selectively depotentiate excitatory inputs on D1-expressing medium spiny neurons, and found a reversal of synaptic plasticity and enduring abolishment of behavioral sensitization to cocaine [88]. The strategy of combining DBS with pharmacology is also novel and may enable precise targeting and modulation of neuroplasticity in key brain regions and networks involved in AD and PD.

Different brain stimulation tools can be combined for both research and clinical purposes. For example, repeated pairing of DBS-TMS pulses at certain time intervals can induce cortical plasticity in PD patients [86]. Also, prior application of tDCS/tACS can potentiate or suppress the rTMS-induced plasticity [89, 90]. Furthermore, patterned DBS and TMS delivered in a repetitive mode are promising novel therapeutic interventions for neurodegenerative diseases.

## Conclusions

The various brain stimulation techniques discussed herein have been found valuable as a research tool, but are not yet suitable as a clinical tool that assists in diagnosis, treatment, or prognosis of individual patients with AD or PD, except the DBS for PD. Well-controlled, translational, and interdisciplinary preclinical and clinical studies are needed for translating basic scientific knowledge into improved diagnostics and therapeutics. To move forward the field of brain stimulation, it is critical to elucidate the specific mechanisms of brain plasticity produced by different brain stimulation techniques, and to optimize the clinical procedure for individualized treatment based on neuroplasticity measurements. The next-generation neuromodulation systems are expected to be more flexible in terms of stimulation parameters and patterns, allowing increased control of stimulation parameters and rapid response to the patient’s ongoing neural activity in a closed-loop manner. Taken together, these research developments and technological innovations hold tremendous promise for improving the safety, clinical efficacy, and diagnostic accuracy of brain stimulation tools for AD and PD patients.

## Data Availability

The datasets used and/or analyzed during the current study are available from the corresponding author on reasonable request.

## Abbreviations

AD: Alzheimer’s disease
DBS: deep brain stimulation
DLPFC: dorsolateral prefrontal cortex
EEG: electroencephalogram
GABA-A: A type γ-aminobutyric acid receptor
GPi: globus pallidus internus
HFS: high-frequency stimulation
LFP: local field potential
LTD: long-term depression
LTP: long-term potentiation
MEP: motor evoked potential
NMDAR: *N*-methyl-D-aspartic acid receptor
PAS: paired associative-stimulation
PD: Parkinson’s disease
SAI: short latency afferent inhibition
SICF: short intracortical facilitation
SICI: short intracortical inhibition
STN: subthalamic nucleus
tACS: transcranial alternating current stimulation
TBS: Theta burst stimulation
tDCS: transcranial direct current stimulation
TEP: TMS evoked potential
TMS: transcranial magnetic stimulation
